# Ontario Neurodegenerative Disease Research Initiative (ONDRI): Structural MRI Methods and Outcome Measures

**DOI:** 10.3389/fneur.2020.00847

**Published:** 2020-08-11

**Authors:** Joel Ramirez, Melissa F. Holmes, Christopher J. M. Scott, Miracle Ozzoude, Sabrina Adamo, Gregory M. Szilagyi, Maged Goubran, Fuqiang Gao, Stephen R. Arnott, Jane M. Lawrence-Dewar, Derek Beaton, Stephen C. Strother, Douglas P. Munoz, Mario Masellis, Richard H. Swartz, Robert Bartha, Sean Symons, Sandra E. Black, The ONDRI Investigators

**Affiliations:** ^1^Hurvitz Brain Sciences Program, Sunnybrook Research Institute, University of Toronto, Toronto, ON, Canada; ^2^Department of Medical Biophysics, University of Toronto, Toronto, ON, Canada; ^3^Rotman Research Institute, Baycrest, Toronto, ON, Canada; ^4^Thunder Bay Regional Health Research Institute, Thunder Bay, ON, Canada; ^5^Centre for Neuroscience Studies, Queen's University, Kingston, ON, Canada; ^6^Department of Medicine (Neurology), Sunnybrook Health Sciences Centre and University of Toronto, Toronto, ON, Canada; ^7^Department of Medical Biophysics, Centre for Functional and Metabolic Mapping, Robarts Research Institute, University of Western Ontario, London, ON, Canada; ^8^Department of Medical Imaging, University of Toronto, Sunnybrook Health Sciences Centre, Toronto, ON, Canada

**Keywords:** MRI, Alzheimer, Parkinson, amyotrophic lateral sclerosis, frontotemporal dementia, cerebrovascular disease, stroke, cerebral small vessel disease

## Abstract

The Ontario Neurodegenerative Research Initiative (ONDRI) is a 3 years multi-site prospective cohort study that has acquired comprehensive multiple assessment platform data, including 3T structural MRI, from neurodegenerative patients with Alzheimer's disease, mild cognitive impairment, Parkinson's disease, amyotrophic lateral sclerosis, frontotemporal dementia, and cerebrovascular disease. This heterogeneous cross-section of patients with complex neurodegenerative and neurovascular pathologies pose significant challenges for standard neuroimaging tools. To effectively quantify regional measures of normal and pathological brain tissue volumes, the ONDRI neuroimaging platform implemented a semi-automated MRI processing pipeline that was able to address many of the challenges resulting from this heterogeneity. The purpose of this paper is to serve as a reference and conceptual overview of the comprehensive neuroimaging pipeline used to generate regional brain tissue volumes and neurovascular marker data that will be made publicly available online.

## Introduction

The Ontario Neurodegenerative Research Initiative (ONDRI) is a multi-site prospective cohort study following patients with neurodegenerative diseases including Alzheimer's disease (AD), mild cognitive impairment (MCI), Parkinson's disease (PD), amyotrophic lateral sclerosis (ALS), frontotemporal dementia (FTD), and cerebrovascular disease (CVD). Over the course of 3 years, multiple assessment platforms acquired comprehensive data from the 520 patients including: neuroimaging (1–3), clinical and demographic assessments (4), neuropsychology (5), genetic variations (6, 7), eye tracking and pupillometry, retinal layer analyses using spectral-domain optical coherence tomography (8), gait and balance performance (9), and neuropathology. The multi-modal data collected from ONDRI will be used to explore earlier detection, guide development of novel therapy, and improve patient care. ONDRI's mission is to bring new diagnostic biomarkers and prognostic tools into clinical practice in order track disease progression and potential response to future symptomatic and disease-modifying therapies targeting dementia/cognitive impairment.

This paper describes the methods implemented to extract normal and pathological brain tissue volumetric information from the structural Magnetic Resonance Imaging (MRI) provided by the ONDRI neuroimaging platform. It includes a comprehensive methodological overview of the structural neuroimaging pipeline's previously published and validated components, with numerous figures to provide a visual description of how the measures were obtained from the MRI, some recommendations for reporting and data analysis, and a brief section providing some basic descriptive statistics to illustrate the whole brain volumetrics that can be obtained from the ONDRI patient cohorts.

**Figure 1:**
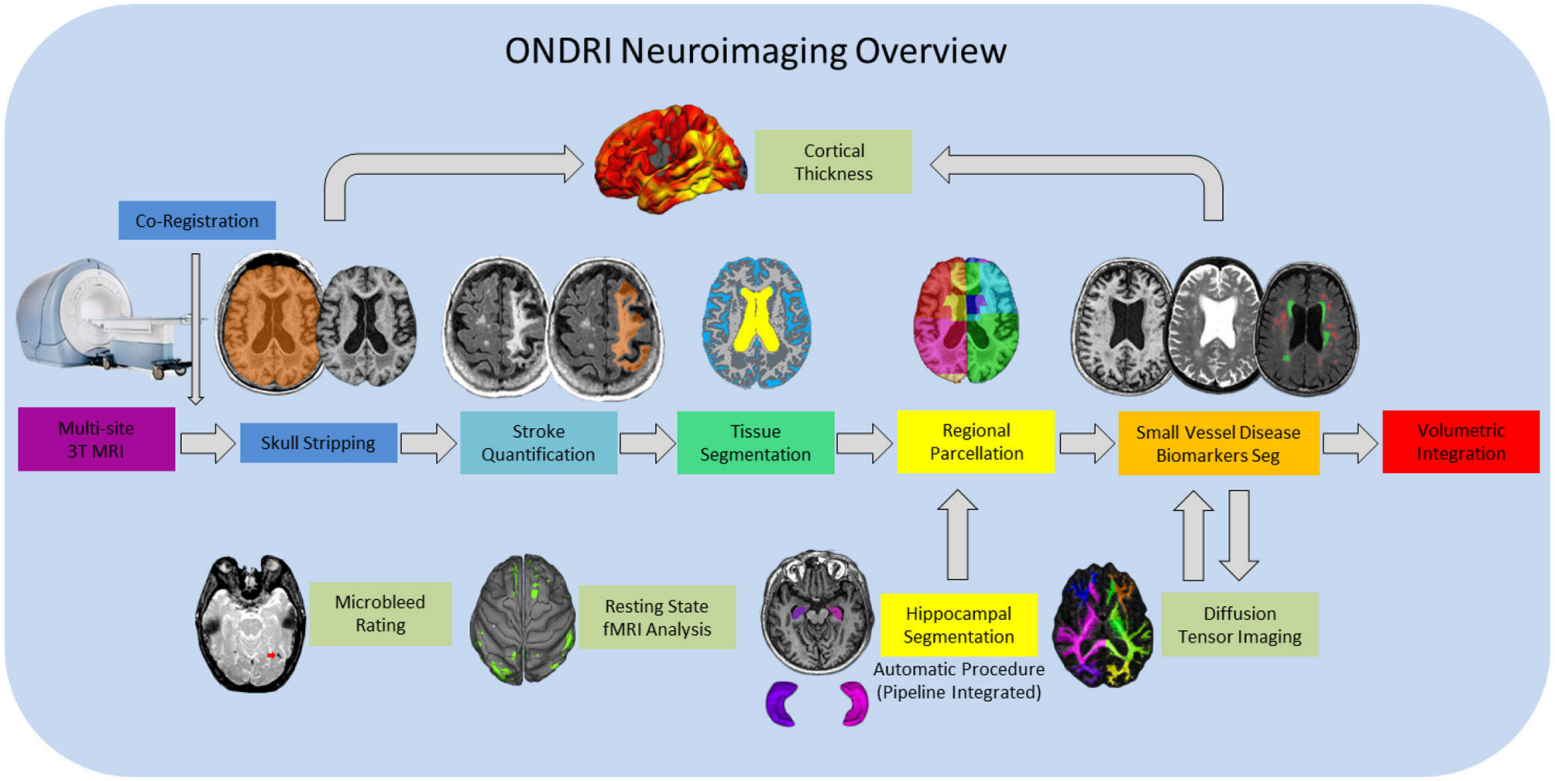
ONDRI MRI processing pipeline overview. General workflow moves from left to right for final volumetric output resulting in a comprehensive spreadsheet in the form of a .csv file. Hippocampal volumes are segmented using the SBHV method (10) which was fully integrated into the pipeline and are included in the final volumetric spreadsheet. Microbleed Rating, Resting State fMRI Analysis, and Diffusion Tensor Imaging (DTI) analyses are processed separately, however, the DTI and Cortical Thickness pipelines are dependent on some components of the primary pipeline, thus, results from these processes are provided in separate spreadsheets.

Structural MRI processing for volumetrics was performed by the neuroimaging group in the L.C. Campbell Cognitive Neurology Research Unit, within the Hurvitz Brain Sciences Research Program, at the Sunnybrook Research Institute, in Toronto, Canada. The image processing pipeline (Figure 1) has been optimized for an aging population, with a particular emphasis on accounting for chronic stroke and post stroke cortical and subcortical lesions, numerous imaging markers of cerebral small vessel disease, as well as, the focal and global brain atrophy observed in neurodegenerative patient populations such as AD and FTD.

The main goal of this paper is to highlight the overall features of the neuroimaging pipeline that would be of interest to a neurologist, clinician, or non-imaging researcher seeking to utilize the ONDRI data that will be made publicly available through an application process on October, 2020. For more information on the ONDRI project, please visit: http://ondri.ca/.

## Methods

### Study Participants

Ethics approval was obtained from all participating institutions. Participants were recruited at 14 health centers across six cities in Ontario, Canada: Hamilton General Hospital and McMaster Medical Centre in Hamilton; Hotel Dieu Hospital and Providence Care Hospital in Kingston; London Health Science Centre and Parkwood Institute in London; Elizabeth Bruyère Hospital and The Ottawa Hospital in Ottawa; Thunder Bay Regional Health Sciences Centre in Thunder Bay; and Baycrest Health Sciences (Baycrest), Centre for Addiction and Mental Health (CAMH), St. Michael's Hospital (SMH), Sunnybrook Health Sciences Centre (Sunnybrook), and Toronto Western Hospital—University Health Network (UHN) in Toronto.

Full study participant details are previously described (4). Briefly, AD/MCI patients met National Institute on Aging Alzheimer's Association criteria for probable or possible AD, or MCI (11, 12); PD patients met criteria for idiopathic PD defined by the United Kingdom's Parkinson's Disease Society Brain Bank clinical diagnostic criteria (13); ALS patients met El Escorial World Federation of Neurology diagnostic criteria for possible, probable, or definite familial or sporadic ALS (14); FTD patients included possible or probable behavioral variants of frontotemporal degeneration (15), agrammatic/non-fluent and semantic variants of primary progressive aphasia (16), and possible or probable progressive supranuclear palsy (17); CVD patients experienced a mild to moderate ischemic stroke event, verified on neuroimaging, 3 or more months prior to enrollment in compliance with the National Institute of Neurological Disorders and Stroke-Canadian Stroke Network vascular cognitive impairment harmonization standards (18).

For illustrative purposes of the neuroimaging pipeline outputs, baseline MRI data are included for the following ONDRI patient cohorts: 126 AD/MCI, 140 PD, 40 ALS, 53 FTD, and 161 CVD.

### MRI Acquisition

Neuroimaging was acquired at the following sites using each site's respective 3T MRI system: a General Electric (GE, Milwaukee, WI) Discovery 750 was used at Sunnybrook, McMaster University/Hamilton General Hospital, and CAMH; a GE Signa HDxt at UHN; a Philips Medical Systems (Philips, Best, Netherlands) Achieva system at Thunder Bay Regional Health Sciences Centre; a Siemens Health Care (Siemens, Erlangen, Germany) Prisma at Sunnybrook and London Health Sciences Centre/Parkwood Hospital; a Siemens TrioTim at Ottawa Hospital/Élisabeth Bruyère Hospital, Hotel Dieu Hospital/Providence Care Hospital and Baycrest; and a Siemens Skyra at SMH.

Harmonized with the Canadian Dementia Imaging Protocol (19), the National Institute of Neurological Disorders and Stroke–Canadian Stroke Network Vascular Cognitive Impairment Harmonization Standards (18), full MRI acquisition protocol details for each imaging site are provided on Supplementary Table 1. In brief, the following structural MRI sequences were obtained for each study participant: 3D T1-weighted (T1), T2-weighted fluid attenuated inversion recovery (FLAIR), interleaved T2-weighted and proton density (T2/PD), and T2^*^gradient recalled echo (GRE). It should be noted that additional imaging protocol included a 30/32 direction diffusion tensor imaging (DTI), resting state functional MRI, and arterial spin labeling (acquired only at one site), but are beyond the scope of this paper and will be presented elsewhere (1). Prior to image processing for volumetric quantification, MRI were fully evaluated by a neuroradiologist (SS) for incidental findings and for imaging quality by a medical biophysics scientist (RB).

### Structural Image Processing Methods: Overview

The structural neuroimaging pipeline used in ONDRI is a component based algorithm commonly referred to as SABRE-Lesion Explorer (SABRE-LE) (20–23). This is a semi-automated personalized approach to imaging-based quantification, as it can provide a comprehensive volumetric profile at the individual patient level. While it may take longer to process each individual relative to fully automatic methods, this careful patient-focused approach is more robust to the large variability in stroke and neurodegenerative patient population. This method has been previously validated (23–25) and implemented in other Canadian studies (26–29). The following sections describe the SABRE-LE comprehensive pipeline method and the volumetric data that is extracted in greater detail. Data visualization was performed using RStudio version 1.2.1335 (RStudio, Inc., Boston, MA) and ITKSnap (30).

### Brain Regions of Interest: SABRE

**Figure 2:**
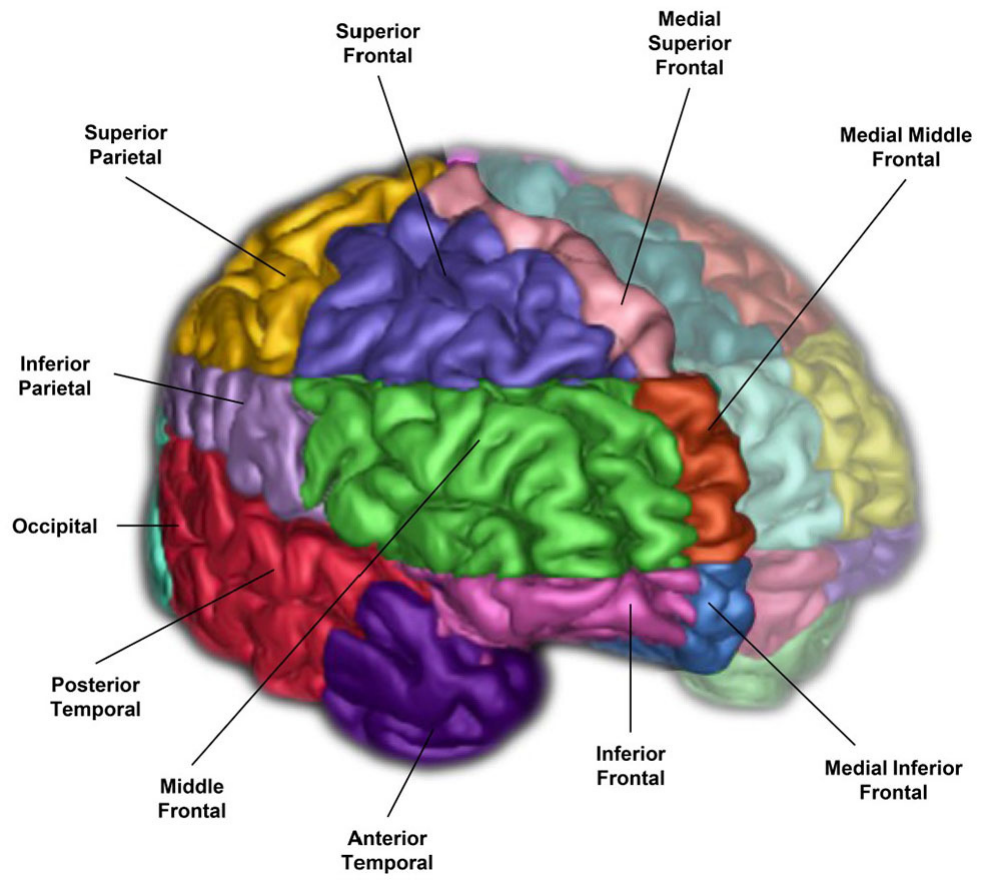
A 3-D surface volume rendering of T1-weighted MRI showing right hemisphere SABRE regions in different colours. Left hemisphere regions were made translucent for illustrative purposes, however, SABRE regions are separately parcellated for each hemisphere and delineated using individualized anatomical landmarks for both left and right sides.

**Figure 3:**
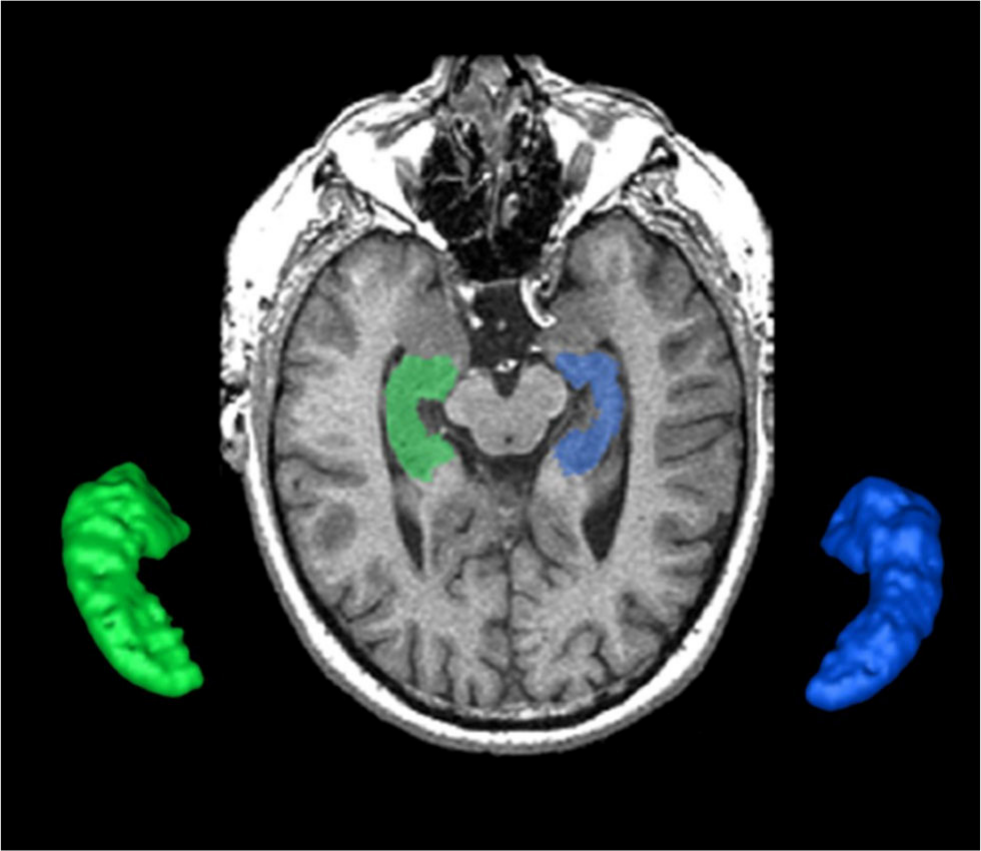
The SunnyBrook Hippocampal Volumetric (SBHV) segmentation showing left (BLUE) and right (GREEN) hippocampi overlayed on an axial T1 MRI and extracted as 3-D surface volume renderings. Note images are in radiological convention.

The neuroimaging pipeline integrates a brain region parcellation process called Semi-Automatic Brain Region Extraction (SABRE) (20). This method separates the brain into 26 regions of interest (ROIs: 13 per hemisphere) derived from anatomical landmarks manually identified per hemisphere on each individual patient (Figure 2 and Table 1). Each imaging analyst was required to achieve an intraclass correlation coefficient (ICC) > 0.90 in order to work on ONDRI patient imaging analysis. The automatic SunnyBrook Hippocampal Volumetry (SBHV) tool (10) was subsequently integrated into the SABRE pipeline (Figure 3), resulting in a total of 28 ROIs (left + right hippocampus) (see following section). The SABRE brain maps are personalized maps that are unique to each individual patient and was developed from the Talairach grid system (31). Relative to many brain mapping methods that implement non-linear (i.e., “warping”) techniques to register an individual patient's MRI to a standardized template, such as the Montreal Neurological Institute brain (MNI152) (32), the SABRE approach is essentially reversed, by mapping a brain template onto the individual patient's MRI. This method accounts for natural individual differences in anatomy but more importantly, it is a method that can compensate for significant focal and global brain atrophy that is found in stroke, dementia, and neurodegenerative patients.

**Table 1:** SABRE-LE neuroimaging pipeline brain tissue and lesion codes (top), and detailed SABRE brain region codes (bottom).

**Imaging descripton**	**Code**	
Supratentorial total intracranial volume	ST_TIV	
Normal appearing gray matter	NAGM	
Normal appearing white matter	NAWM	
Sulcal cerebrospinal fluid	CSF	
Ventricular cerebropsinal fluid	CSF	
Periventricular white matter hyperintensities	Pwmh	
Deep white matter hyperintensities	dWMH	
Periventricular lacunes	pLACN	
Deep lacunes	dLACN	
Enlarged perivascular spaces	PVS	
Chronic stroke lesions	Stroke	
**SABRE brain region name**	**Code**	**Lobe**
Superior frontal	SF	Frontal
Middle frontal	MF	Frontal
Inferior frontal	IF	Frontal
Medial inferior frontal	MIF	Frontal
Medial superior frontal	MSF	Frontal
Medial middle frontal	MMF	Frontal
Superior parietal	SP	Parietal
Inferior parietal	IP	Parietal
Occipital	O	Occipital
Anterior temporal	AT	Temporal
Posterior temporal	PT	Temporal
Anterior basal ganglia/thalamus	ABGT	Basal ganglia/thalamus
Posterior basal ganglia/thalamus	PBGT	Basal ganglia/thalamus
Hippocampus	HP	Medial temporal

*Note that each regional code will be preceded by an “L” or “R” indicating the left or right hemisphere*.

#### Hippocampus

The hippocampus is an important part of the limbic system that has been studied extensively in dementia, given its significant role in memory functions (33, 34). The ONDRI pipeline incorporates the multi-atlas based Sunnybrook Hippocampal Volumetric (SBHV) segmentation tool (Figure 3) that was developed and validated using the Sunnybrook Dementia Study and the Alzheimer's Disease Neuroimaging Initiative (ADNI1) (10).

For ONDRI, the SBHV segmentation has been fully integrated into the SABRE-LE pipeline, and includes left and right hippocampal sub-classifications for parenchyma, hypointensities, and stroke volumes (when present). Currently, there is some controversy over the pathophysiological origin and relevance of small cavities commonly observed in the hippocampus (35–38), which are particularly relevant in the ONDRI CVD patients. Additionally, large cortico-subcortical strokes can extend from the cortex into the hippocampus. Given these vascular issues potentially affecting the overall hippocampal volume, ONDRI provides sub-classifications for parenchyma, hypointensities, and stroke volumes based on the neuroimaging characteristics (i.e., intensity) using the voxel segmentation classifications and takes a neutral stance on the pathophysiological origin of small cavities observed in this region.

### Total Intracranial Volume

The supratentorial total intracranial volume (ST-TIV) is a measure of all brain matter that is located below the dura mater. It is referred to as *supratentorial* because the SABRE-LE method removes all tissue below the tentorium, including the cerebellum and portions of the brain stem (20, 22). Although the removal of infratentorial structures was necessary for technical segmentation reasons, researchers particularly interested in the cerebellum, and brainstem can apply additional imaging tools [e.g., (39)] to obtain these structures from the original acquisitions upon special request.

**Figure 4:**
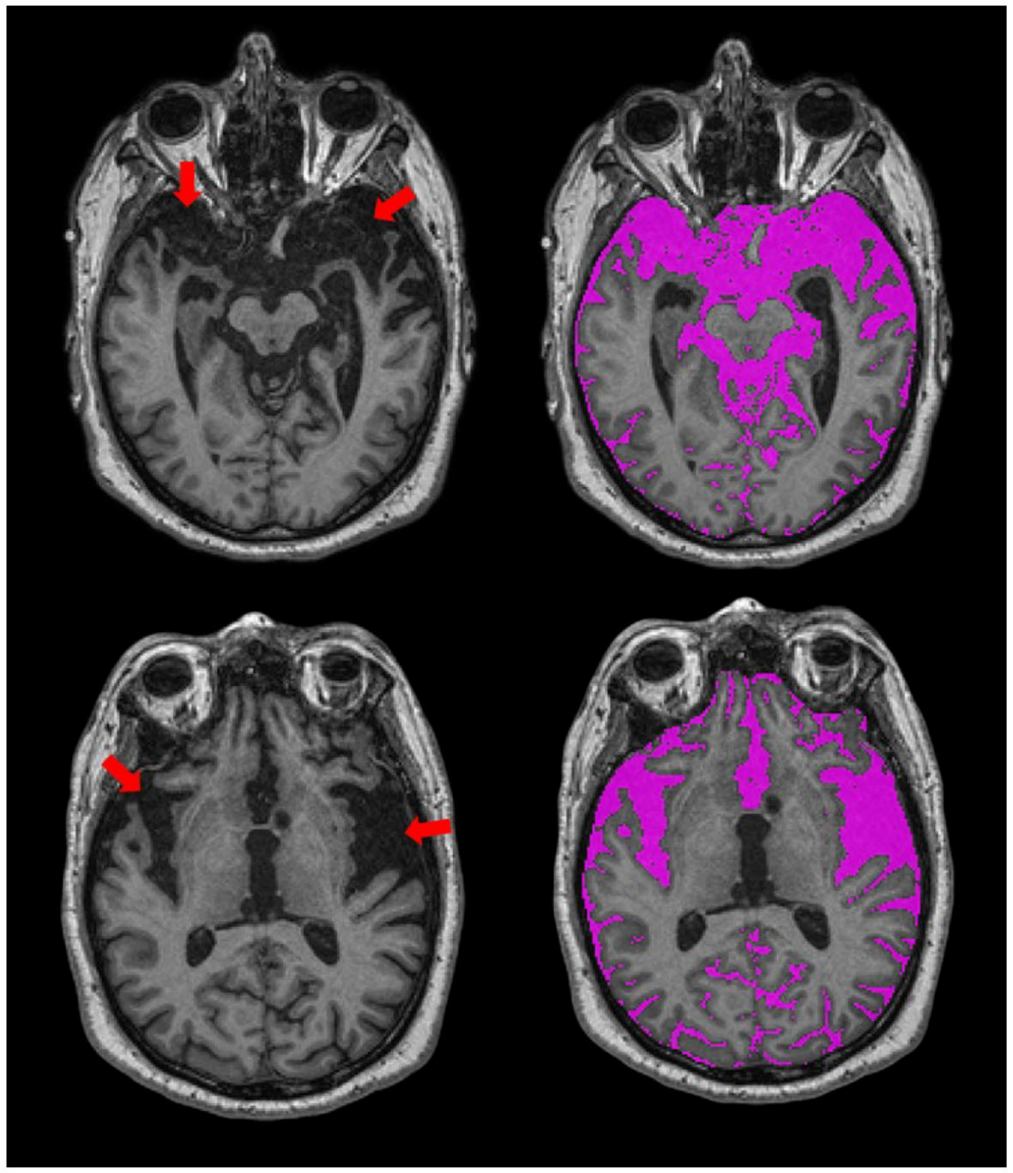
Axial views of T1-weighted MRI from an ONDRI FTD patient. Red arrows point to regions with significant focal brain atrophy. The SABRE-LE processing pipeline accounts for this focal atrophy since it includes a measure of everything below the dura mater, including sub-arachnoid and sulcal cerebrospinal fluid (CSF), shown in purple.

In addition to sex-related differences, there are also normal variations in head size. In order to account for these differences, most neuroimaging studies implement some form of head-size correction. This is also particularly important when assessing brain atrophy in cross-sectional studies, as a true measure of the total intracranial capacity will provide an indication of where “there used to be brain and now there is cerebrospinal fluid (CSF).” The presence of focal atrophy due to stroke and neurodegenerative processes tends to result in over and under erosion errors with many fully automated T1-based skull stripping techniques, due to the similarity in intensity between background and sulcal CSF. The SABRE-LE method accounts for the presence of focal atrophy since it includes a measure of everything below the dura mater, including sub-arachnoid CSF, thus, providing a more accurate measure of head-size in neurodegenerative patient populations (Figure 4).

It is important to note that there are numerous acceptable head-size correction methods reported in the literature (40). A simple method involves dividing each volume of interest by the total head size to obtain a proportional volume (41). ONDRI provides raw volumes and head size volumes (i.e., ST-TIV) for each individual patient.

### Brain Tissue Segmentation

A robust T1 intensity-based brain tissue segmentation, optimized for aging and dementia, is performed after skull stripping and removal of non-brain tissue (24). This automatic segmentation method deals with scanner inhomogeneities by fitting localized histograms to Gaussians to allocate voxels into gray matter (GM), white matter (WM), and cerebrospinal fluid (CSF) tissue classes. After manual ventricular CSF (vCSF) relabelling, there are four brain tissue types that are segmented for volumetrics using SABRE-LE (Table 2):

- Normal appearing gray matter (NAGM)
- Normal appearing white matter (NAWM)
- Sulcal cerebrospinal Fluid (sCSF)
- Ventricular CSF (vCSF).

**Table 2:** Data is shown as mean (standard deviation) unless otherwise specified. Raw values are presented for transparency purposes.

**Demographics**	**AD/MCI**	**ALS**	**FTD**	**PD**	**CVD**
Number of participants	126	40	52	140	155
Age, years	71.0 (8.2)	62.0 (8.7)	67.8 (7.1)	67.9 (6.3)	69.3 (7.4)
Sex, *n* (%) female	57 (45.2)	16 (40.0)	19 (36.5)	31 (22.1)	48 (31.0)
ST-TIV, cc	1235.6 (144.6)	1203.6 (162.8)	1245.8 (129.6)	1316.6 (127.0)	1224.5 (133.2)
NAWM, cc	395.4 (344.5)	425.0 (78.8)	295.1 (59.4)	446.1 (61.2)	387.4 (54.4)
NAGM, cc	533.3 (51.4)	556.2 (65.7)	252.5 (56.0)	574.7 (47.1)	535.7 (52.3)
sCSF, cc	256.3 (62.1)	195.9 (52.9)	277.0 (57.8)	252.3 (53.3)	242.6 (59.3)
vCSF, cc	45.7 (28.4)	23.8 (11.1)	43.7 (16.6)	38.2 (19.4)	41.3 (23.0)
pWMH^*^, mm^3^	2564.5 (2811.2)	1040.0 (1252.5)	2736.0 (1623.8)	2563.5 (2708.0)	4054.0 (7468.0)
dWMH^*^, mm^3^	289.5 (424.7)	208.0 (386.5)	138.5 (379.3)	259.5 (225.7)	555.0 (584.0)
LACN^*^, mm^3^	15.5 (66.0)	14.5 (12.2)	9.5 (55.5)	17.5 (70.0)	92.0 (291.0)
PVS^*^, mm^3^	45.5 (35.5)	17.5 (9.5)	32.5 (36.3)	34.0 (30.0)	44.0 (33.0)
Stroke^*^, mm^3^	–	90.0^a^	393.0 (294.0)^b^	531.5 (1269.0)^c^	4644.5 (12963.0)^d^

* *Data is shown as median (interquartile range)*.

a *Available in 1/40 participants*.

b *Available in 6/52 participants*.

c *Available in 4/140 participants*.

d *Available in 88/155 participants AD/MCI, Alzheimer's Disease and Mild Cognitive Impairment; ALS, Amyotrophic Lateral Sclerosis; FTD, Frontotemporal Dementia; PD, Parkinson's Disease; VCI, Vascular Cognitive Impairment; ST-TIV, supratentorial total intracranial volume; NAWM, normal appearing white matter; NAGM, normal appearing gray matter; sCSF, sulcal cerebrospinal fluid; vCSF, ventricular cerebrospinal fluid; pWMH, periventricular white matter hyperintensities; dWMH, deep white matter hyperintensities; LACN, lacunes; PVS, perivascular spaces*.

**Figure 5:**
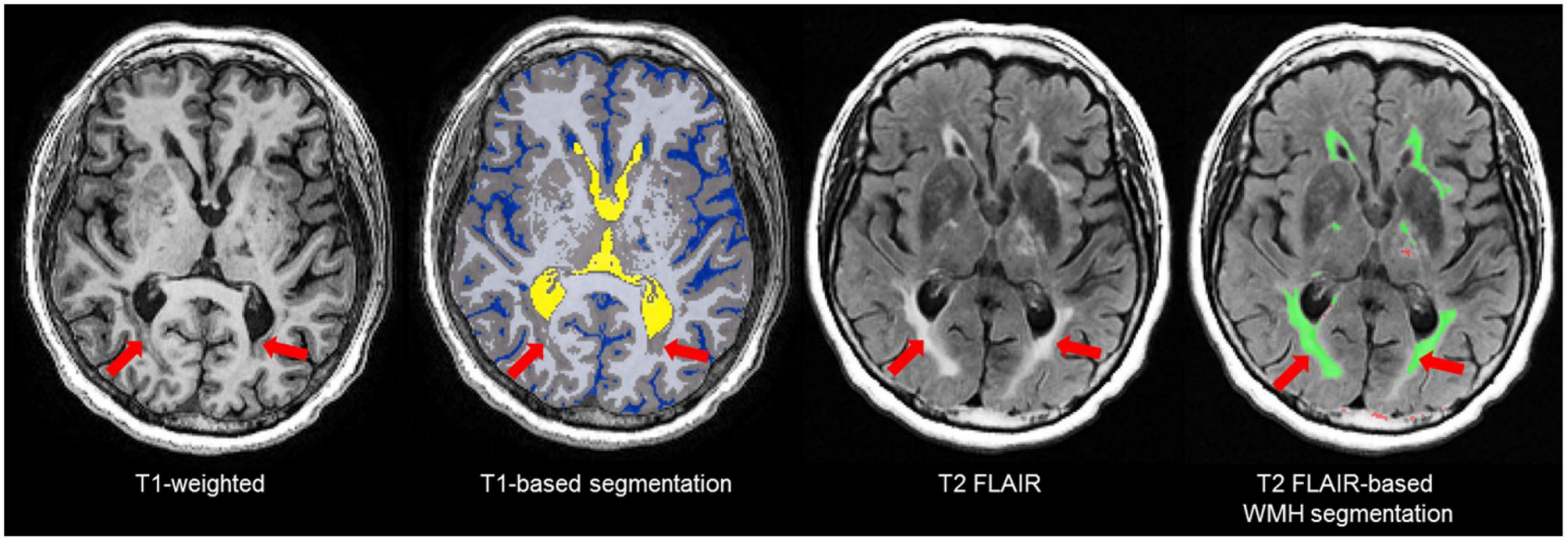
Due to relative intensities on different MRI sequences, WMH (red arrows) on T2 FLAIR are not hyperintense (bright) on T1-weighted images and tend to appear as GM (gray) or CSF (blue) intensity on T1. Thus, T1-based segmentations tend to inflate the GM and CSF volumes in patients with stroke and cerebral small vessel disease. To account for this, ONDRI's imaging pipeline integrates an additional T2/FLAIR-based WMH segmentation to correct for this misclassification error (42) to produce a normal appearing WM/GM (NAWM/NAGM) volumes.

The T1-based tissue segmentation is further corrected for misclassified volumes using a PD-T2/FLAIR-based lesion segmentation algorithm to account for the voxels appearing as GM or CSF on T1 (42) due to WM changes from stroke and cerebral small vessel disease. For this reason, the GM and WM volumes are denoted as “normal appearing” (NAGM, NAWM) to signify that these volumes have been re-labeled as normal appearing after having been corrected with an additional multi-modal MRI segmentation approach (Figure 5). Additional brain tissue volumes for stroke lesions and cerebral small vessel disease markers are discussed in the following sections.

The NAGM and NAWM volumes can be summed to obtain a measure of parenchymal volume or reported individually for head-size corrected measures to assess potential atrophy. Additionally, a segmentation mask is generated which is used for diffusion tensor imaging (DTI) analyses, where diffusion metrics of the “normal appearing” WM tracts can be separately analyzed from the diffusion within the various types of white matter lesions including WMH, lacunar infarcts, and cortical-subcortical stroke lesions. Details of ONDRI DTI analysis pipeline are discussed elsewhere (1).

**Figure 6:**
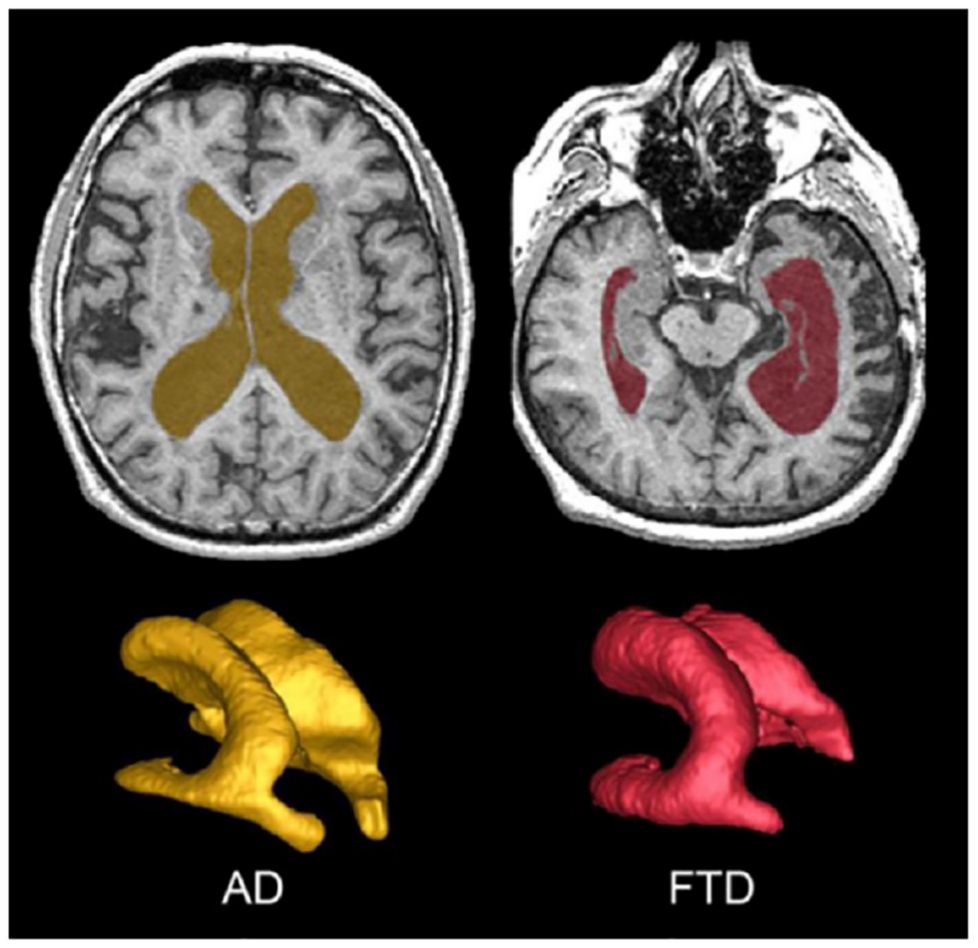
Top row shows axial view of vCSF segmentation overlayed on T1 MRI for patients with AD (left) and FTD (right). Bottom row shows 3D surface volume renderings of the vCSF segmentation. Note the differences in ventricle size and the hemispheric differences between the two neurodegenerative diseases.

The SABRE-LE method segments sCSF and vCSF into separate compartments. The initial T1-based segmentation automatically labels hypointense voxels into a CSF class, and then the ventricles are manually relabelled to a vCSF class by neuroimaging analysts following a standardized procedure. Note that although some vCSF segmentation tools based on standardized templates use smoothing algorithms that reclassify all voxels within the ventricular compartment as ventricles, the SABRE-LE method does not. With the SABRE-LE method, choroid plexus are not arbitrarily removed or re-classified as CSF and thus remain as part of the overall tissue segmentation. Ventricular volumes are often used as a simple indicator of overall brain atrophy, and have the potential for use as a differential indicator of disease and dementia severity (Figure 6) (43–45).

### White Matter Hyperintensities of Presumed Vascular Origin (WMH)

**Figure 7:**
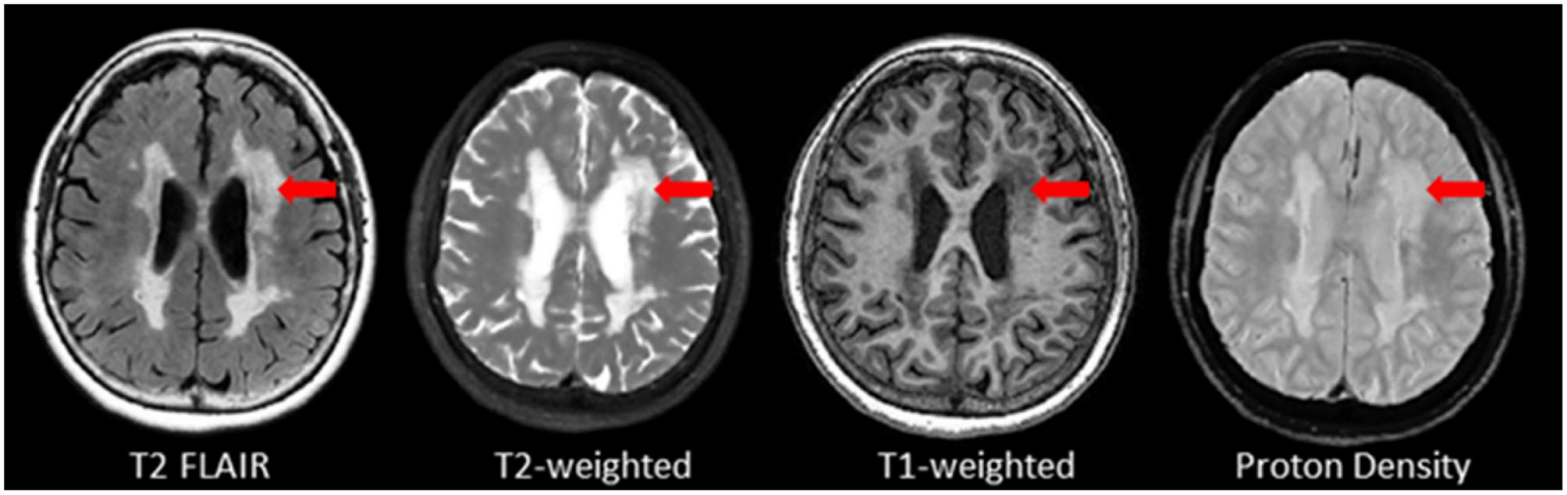
Axial view of various coregistered structural MRI sequences showing the relative intensity differences of WMH. Note that white matter hyperintensities are not hyperintense (i.e., bright) on T1-weighted MRI.

Also referred to as leukoaraiosis, white matter lesions, subcortical hyperintensities, and even, unidentified bright objects, WMH are radiological anomalies commonly associated with cerebral small vessel disease. Recently, the STandards for ReportIng Vascular changes on nEuroimaging (STRIVE) (46) have established a set of criteria that recommends the use of the term *white matter hyperintensities of presumed vascular origin* (WMH), as the standard terminology to refer to these regions of hyperintense (bright) signal found on particular MRI. It is important to note that as previously mentioned, WMH do not appear hyperintense on all types of MRIs and often appear isointense to GM on T1 (Figure 7). Additionally, despite the naming convention, it is important to note that WMH are not limited to the white matter regions of the brain, as they are also commonly observed in subcortical GM structures such as the basal ganglia and thalamus. However, to avoid confusion between studies, ONDRI recommends the use of the more popular term “white matter hyperintensities.”

#### Periventricular (pWMH) and Deep White (dWMH) Hyperintensities

**Figure 8:**
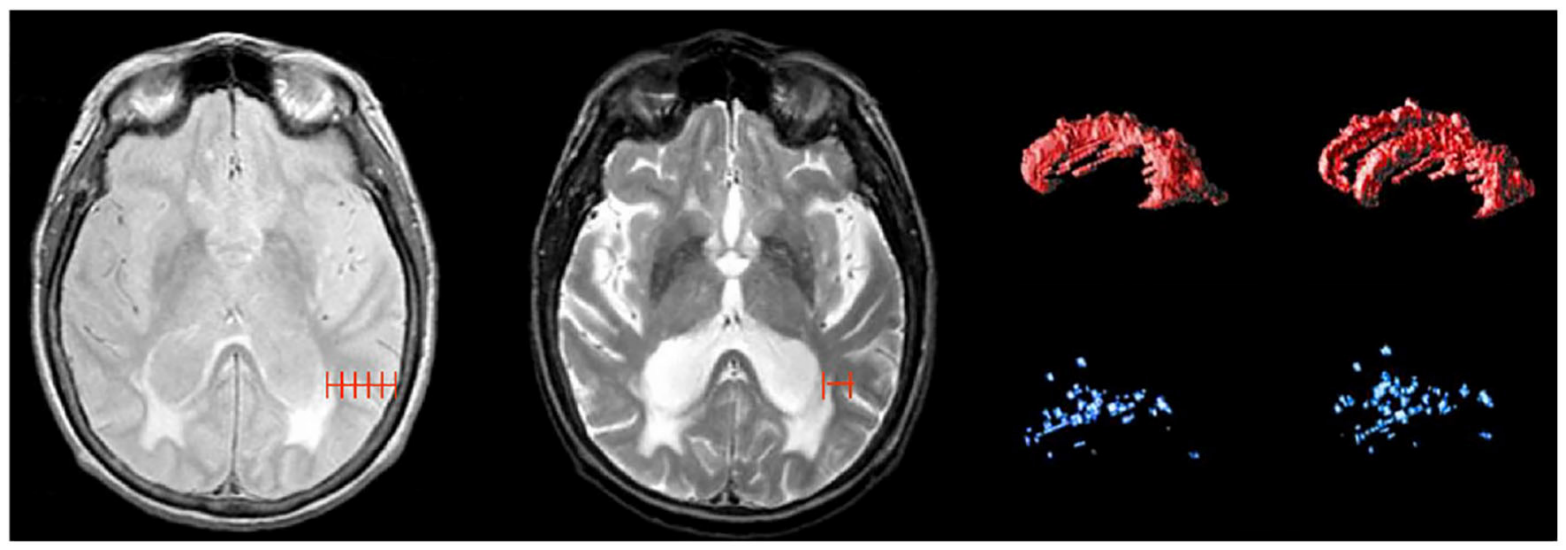
Shows different methods for segmenting periventricular and deep WMH. Left image shows a proportional distance from the ventricular lining to the dura mater; middle image shows an arbitrary distance of 13 mm from ventricles, right image shows 3D connectivity algorithm supported by ONDRI, displayed as 3D volume renderings of pWMH (red) and dWMH (blue) shown in sagittal and slightly tilted anterior views.

Although WMH can be subdivided using SABRE ROIs, the most common regional delineation of WMH is the separation between periventricular (pWMH) and deep white (dWMH). Historically controversial (47, 48), this concept is based on several theories and research findings which suggest that WMH in close proximity to the ventricles (hence the term “peri-ventricular”) have a different pathological etiology (49, 50) and are differentially correlated with cognitive/behavior deficits in comparison to the more distal dWMH (despite the confusing fact that pWMH are technically found in deeper white matter than dWMH). Additionally, recent imaging-pathology correlations suggest that a common substrate of pWMH relates to vasogenic edema due to leakage and increased vascular resistance caused by venous collagenosis, a small vessel venular disease of the deep medullary venules (as opposed to the arterial side of the cerebral vasculature) (51–53). It is also interesting to note that there is no standard consensus in the literature on how to define pWMH vs. dWMH, with some papers using a proportional distance to the dura mater (54), some using an arbitrary cut-off (typically 13 mm from the ventricles) (55), and others using a 3D connectivity algorithm (23, 56)—the method that is currently supported by ONDRI (see Figure 8).

### Lacunes

**Figure 9:**
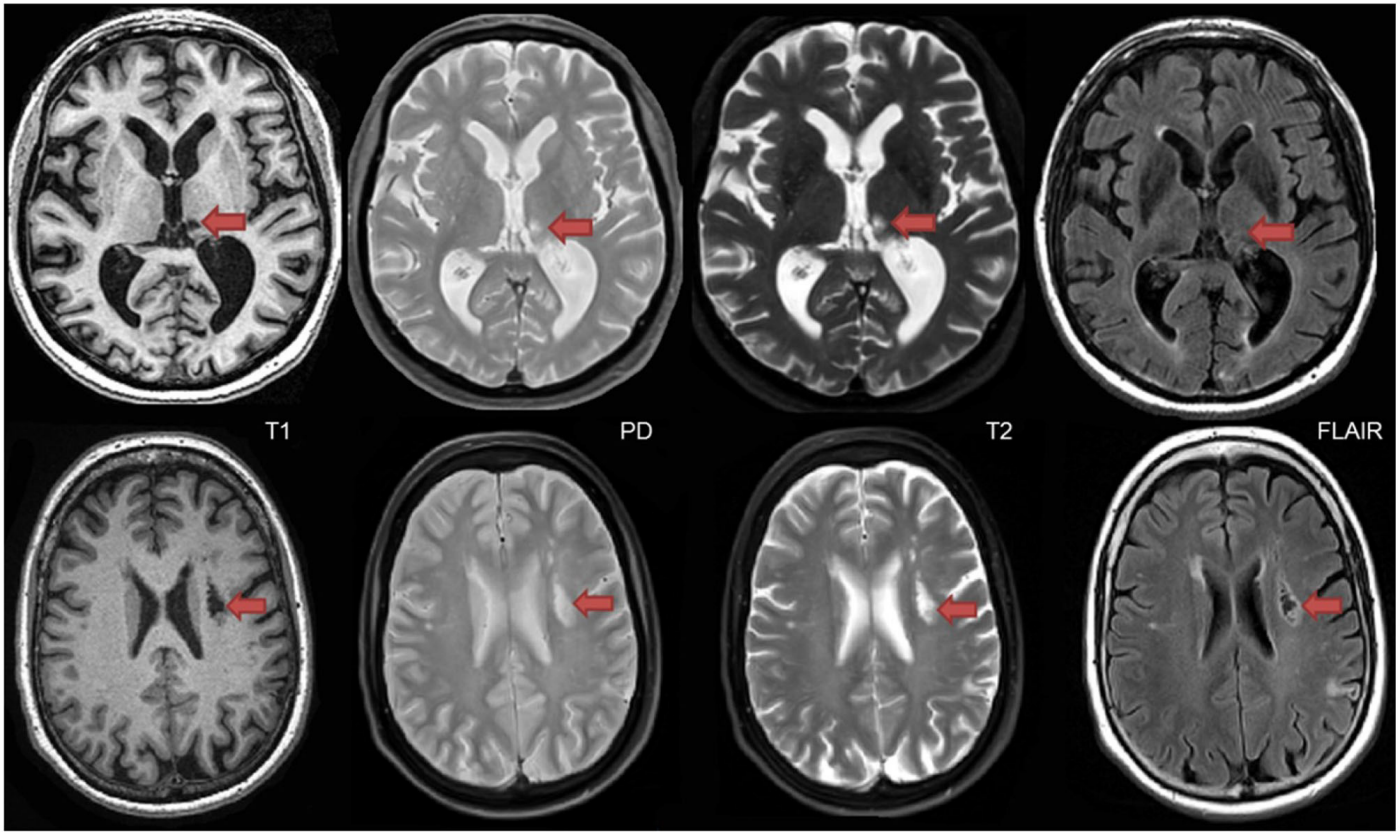
Top row shows a thalamic lacune as it appears on different coregistered MRI, hypointense (dark) on T1, hyperintense on PD-T2, and difficult to detect on FLAIR. In contrast, the bottom row shows a subcortical lacunar infarct that presents with the classic central CSF-like hypointensity with a surrounding hyperintense halo/rim on FLAIR.

Lacunes of presumed vascular origin are cystic fluid-filled cavities in the subcortical brain regions (57, 58). They appear hypointense (dark) on T1, hyperintense (bright) on PD and T2, and can appear as a lesion with a hypointense central core surrounded by a hyperintense rim/halo on FLAIR MRI (Figure 9, bottom row). The recent STRIVE criteria (46) provides some consensus-based guidelines regarding their definition, however, previous studies have used various terms (e.g., “*white matter lesions*,” “*lacunar infarcts*,” “*covert strokes*”) and radiological descriptions to classify these lesions (59). Often difficult to differentiate from MRI-visible perivascular spaces (PVS) (next section), lacunes tend to be larger and less linear than PVS. They are associated with increased risk of stroke, dementia, and gait disturbances (60). It is important to note that due to the poor sensitivity of FLAIR in thalamic regions (61) (Figure 9, top row), the ONDRI imaging pipeline integrates an additional T2-based segmentation in order to capture any potential lesions in this subcortical region that may not appear on FLAIR.

### MRI-Visible (Enlarged) Perivascular Spaces (PVS)

**Figure 10:**
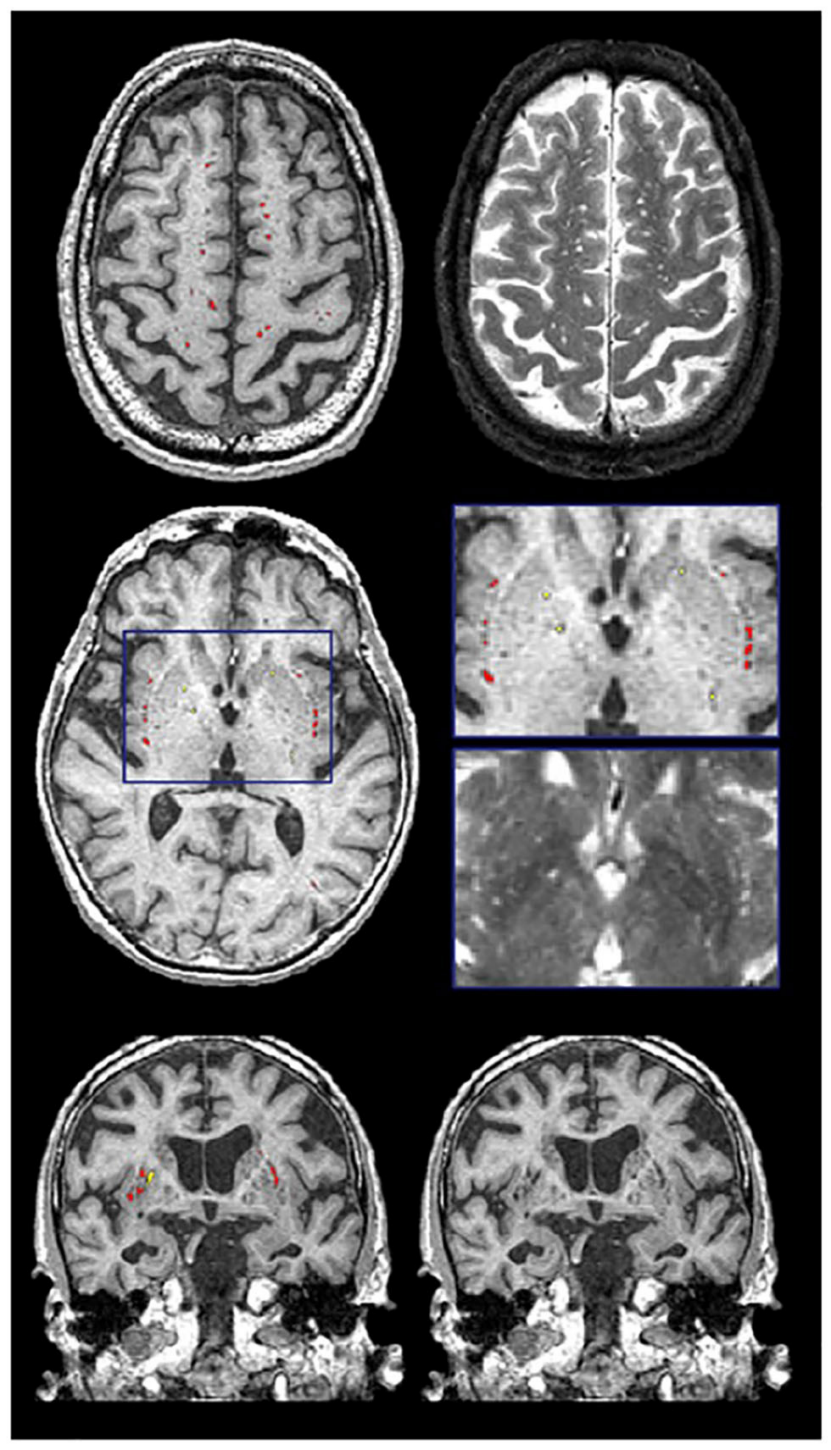
Examples of the PVS segmentation (red and yellow) over-layed onto structural MRI in axial (top 2 rows) and coronal views (bottom row).

Recent studies suggest that the brain utilizes the glymphatic system (62, 63) to clear fluid and metabolic waste, using a complex series of perivascular channels surrounding the brain's veins and arteries. It has been suggested that when the perivascular channels are compromised due to aging, disease, or trauma, the perivascular space becomes enlarged and consequently, visible on structural MRI (64–67). MRI-visible (enlarged) perivascular spaces (PVS) on T2 appear as small (<3 mm diameter), linear, hyperintensities following the course of the vasculature (Figure 10). Additionally, PVS appear hypointense (dark) on T1, isointense to GM on PD (vs. lacunes which are bright on PD), and are very difficult to visualize on 2D FLAIR, particularly in the basal ganglia region. Current research suggests that PVS found in the white matter regions may indicate Cerebral Amyloid Angiopathy (CAA), while PVS in the basal ganglia may be more indicative of hypertensive arteriopathy (68–71). Moreover, recent basic science research and limited clinical evidence supports the theory that clearance of amyloid and other metabolites occurs primarily during deep sleep (72, 73).

Previously referred to as dilated Virchow-Robin spaces, measurement of PVS burden is typically accomplished using visual rating scales under this old naming convention (74, 75). However, the novel quantitative method supported by ONDRI provides a volumetric measure of PVS. This method has been previously validated with common PVS visual scales and has been used to study AD, normal elderly, and stroke and cerebrovascular disease patients being assessed with sleep polysomnography (72, 76). Although both lacunes and PVS volumes are segmented automatically using the SABRE-LE pipeline, false positive minimization procedures are manually performed to remove incorrect segmentations and to reallocate PVS to lacunes or vice versa depending on strict intensity and shape-based criteria. Only highly trained neuroimaging analysts achieving ICCs and DICE Similarity Indices (SI) > 0.90 are allowed to perform this procedure. Moreover, a research neuroradiologist (FG) was consulted when faced with complex radiological anomalies that were commonly observed in the CVD patient cohort.

### Cerebral Microbleeds

**Figure 11:**
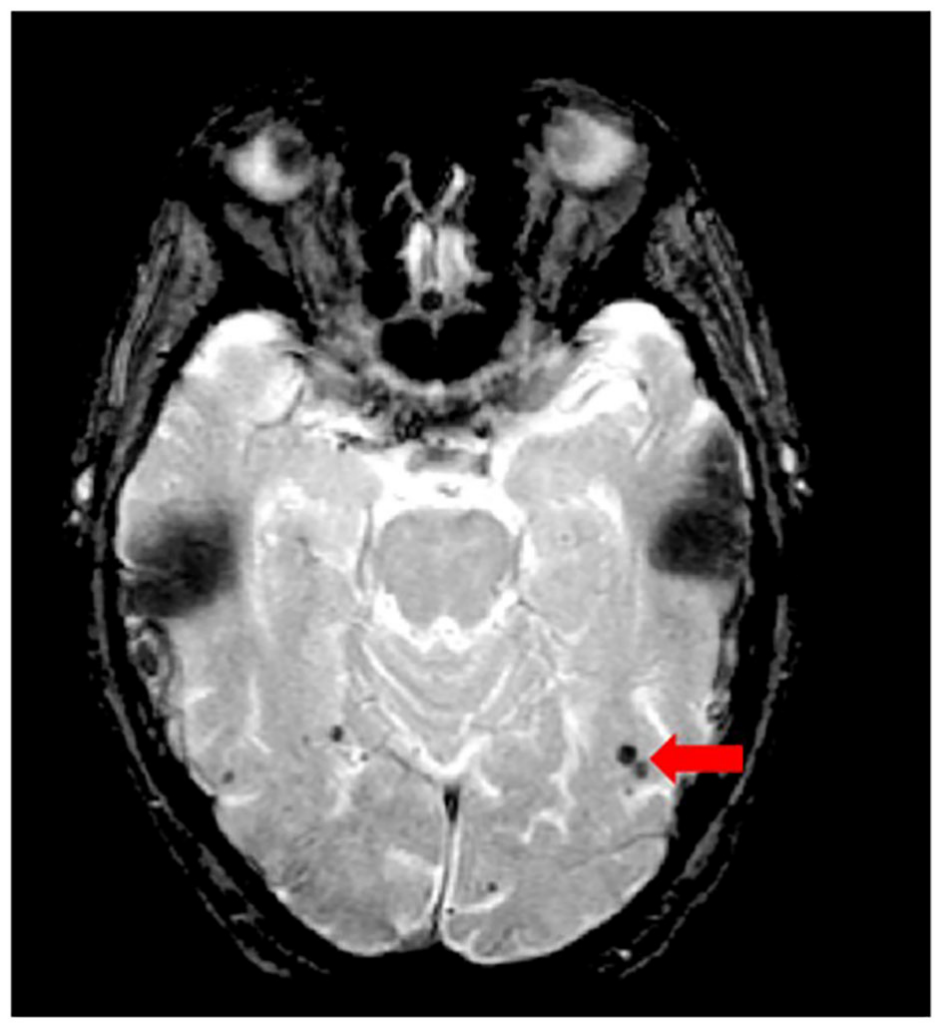
Axial view of iron-sensitive T2* gradient echo (GRE) with red arrow pointing to cerebral microbleeds visualized as hypointensities (dark).

Although the SABRE-LE structural pipeline method used by ONDRI does not support a cerebral microbleed (CMB) segmentation algorithm, this brief section has been included to describe this important measure of cerebral small vessel disease burden. In ONDRI, CMB, and superficial siderosis burden are being assessed visually by a highly qualified neuroradiologist (SS). Cerebral microbleeds (CMB) have been shown to reflect perivascular leakage of red blood cells that can be visualized as low signal intensities (hypointense/dark spots) on T2^*^-weighted gradient-recalled echo (GRE) (Figure 11) and susceptibility weighted imaging (SWI) (77). There are two commonly used methods of assessing CMB burden, the Microbleed Anatomical Rating Scale (MARS) (78) and the Brain Observer MicroBleed Scale (BOMBS) (79) visual rating scales. Previous studies have shown that CMB are associated with an increased risk of stroke, intracerebral hemorrhage, cognitive decline, and dementia (80–84). Differences in anatomical distribution suggest that CMB found in deep centrencephalic brain regions (basal ganglia, thalamus, and brain stem) are more closely related to hypertensive arteriopathy (85), while lobar CMB are more closely associated with CAA and AD pathology (86–89), leading to the development of the Boston criteria for the diagnosis of possible/probable CAA (90, 91).

### Chronic Stroke

According to recent estimates, stroke is the 2nd most common cause of death worldwide (92) and the second leading cause of dementia (93). In a 2013 global report, there were ~25.7 million stroke survivors, and 7.5 million deaths from ischemic and hemorrhagic stroke (94). In Canada, ~62,000 people are treated for stroke and transient ischemic attack. In a series of publications, the Heart and Stroke Foundation Canadian Best Practice Committees have been developing various evidence-based recommendations to address issues regarding: telestroke technologies (95); managing transitions of care following stroke (96); mood, cognition, and fatigue following stroke (97); hyperacute stroke care (98); secondary prevention of stroke (99); and stroke during pregnancy (100, 101).

Although the term “stroke” may encompass a wide range of clinical criteria (102), the Vascular Cognitive Impairment (VCI) (18) inclusion-exclusion criteria for ONDRI CVD patients was limited to mild-moderate ischemic stroke patients, defined by a Modified Rankin Scale (MRS) (103) score of 0–3. It is important to note that although there are a number of imaging techniques used to measure acute stroke in the early stages (within a couple of hours of stroke), the MRI methods applied to ONDRI CVD patients are measures of post-stroke lesions, often referred to as *chronic stroke*, with structural MRI acquired > 3 months post ischemic stroke event.

**Figure 12:**
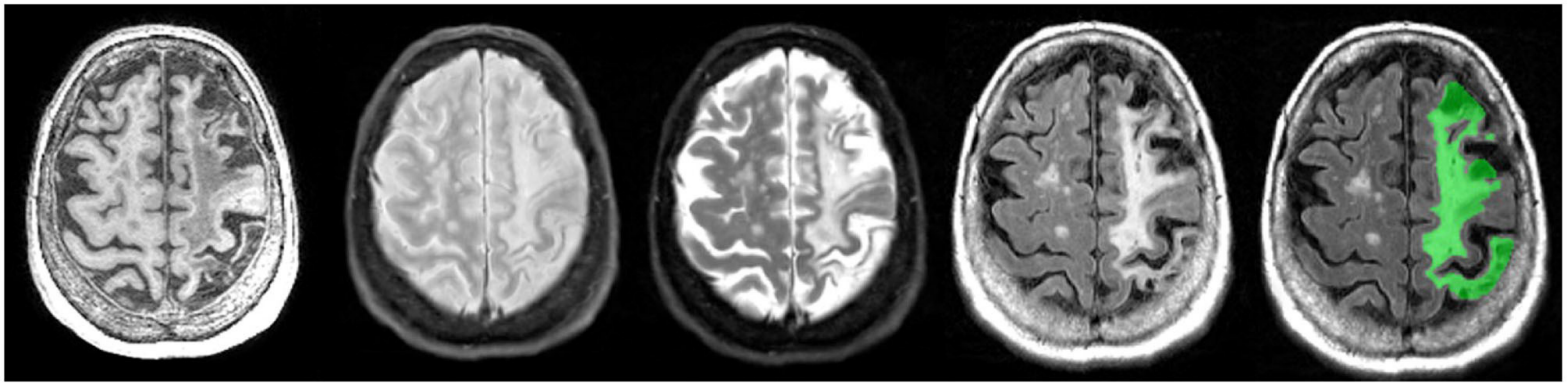
Axial view of coregistered structural MRI sequences (left to right): T1, PD, T2, and FLAIR. Images illustrate relative intensity differences of a large cortico-subcortical stroke lesion across various types of MRI. The last pane shows ONDRI's manual segmentation of the entire stroke core and surrounding hyperintense partially infarcted tissue volume in green.

As there are currently no reliable automatic ways to quantify the range of cortico-subcortical stroke lesions, ONDRI neuroimaging analysts manually delineate the stroke under the direct supervision of a highly experienced research neuroradiologist (FG). This manual delineation is strictly limited to cortical strokes appearing as hyperintense (bright) on FLAIR and hypointense (dark) on T1, although the entire stroke volume often extended into the subcortical regions of the brain (Figure 12). Although this total volume does not separate the hypointense necrotic stroke core from the surrounding partially infarcted hyperintense region indicating varying degrees of gliosis and encephalomalacia, future automatic segmentation techniques are currently being tested in ONDRI to include this sub-segmentation.

## Recommendations for Reporting and Analysis

Here we provide some general guidelines for reporting and analysis that can be useful for researchers wishing to use ONDRI data.

First and foremost, when reporting data for characterization of the sample being analyzed, we recommend that the original raw volumes are reported in tables for transparency and between-study comparisons; however, statistical analyses should generally be performed on head-size corrected volumes. Head size correction accounts for individual variations in intracranial capacity and sex-related differences in head size (104). Additionally, depending on the research question, the volume of interest (i.e., NAWM, NAGM, CSF, or WMH) could also be reported as a proportion of the total volume within each SABRE region, or they can be reported as a proportion of the total head-size (ST-TIV) for age-independent normalization/correction. The version or date of the data release should also be reported.

There are several ways that WMH can be analyzed and it depends on the research question in mind. The simplest approach is to sum the dWMH and pWMH, which results in a whole brain measure of small vessel disease burden. Regional analyses of WMH can also be performed to assess WMH burden within a SABRE ROI. Additionally, WMH within different ROIs can be combined by simply summing the volumes from different SABRE regions to generate a larger ROI (e.g., sum all pWMH and dWMH volumes within all frontal SABRE brain region parcellations using the Frontal Lobe Codes shown on Table 1).

It is important to note that many measures of cerebral small vessel disease, such as pWMH and dWMH, are typically non-normally distributed (105), often inter-correlated (54), are known to be age-related (106), and commonly associated with vascular risk factors such as hypertension (107). Thus, careful attention to these factors and previous research findings highlight ONDRI's recommendation to consider these additional factors when analyzing imaging-based markers of cerebral small vessel disease. Given the skewed, non-normal distribution of WMH (even after head-size correction), WMH volumes are typically transformed (e.g., log) prior to standard parametric analyses. For this reason, approaches designed to deal with complex distributions should be considered (108).

Since the pipeline automatically segments lesions in the periventricular region from the deep white regions, the lacunar volumes are also provided in this manner. While some future studies may argue a pathophysiological difference between these two locations of lacunar presentation, there are currently limited studies to suggest this anatomical delineation. Given this, we recommend that the two volumes be summed together prior to analysis. Interestingly, lacunes and PVS volumes are not typically head-size corrected in the clinical/scientific literature, however, age, sex, WMH, and a measure of brain atrophy (e.g., BPF or vCSF), and proper accounting of vascular risk are recommended covariates when analyzing lacunes and PVS (109, 110). Note that in many publications, lacunes, and PVS are reported as counts (i.e., number of), because they are often measured using visual rating methods that require the user to count the number of lacunes or PVS observed on an MRI—often leading to wide variations in definitions and conflicting findings in the literature (59, 111). Since the lacunes and PVS in ONDRI are quantified using segmentation based imaging analyses, PVS and lacunar volumes are provided rather than counts.

Finally, any analyses using ONDRI's CVD cohort should consider the common comorbidities of depression, obstructive sleep apnea, and cognitive impairment (112), as well the subcortical silent brain infarcts/lacunes, WMH, and potentially, CMB, which have recently been acknowledged as playing an important role in primary stroke prevention (113).

## Results and Conclusion

**Figure 13:**
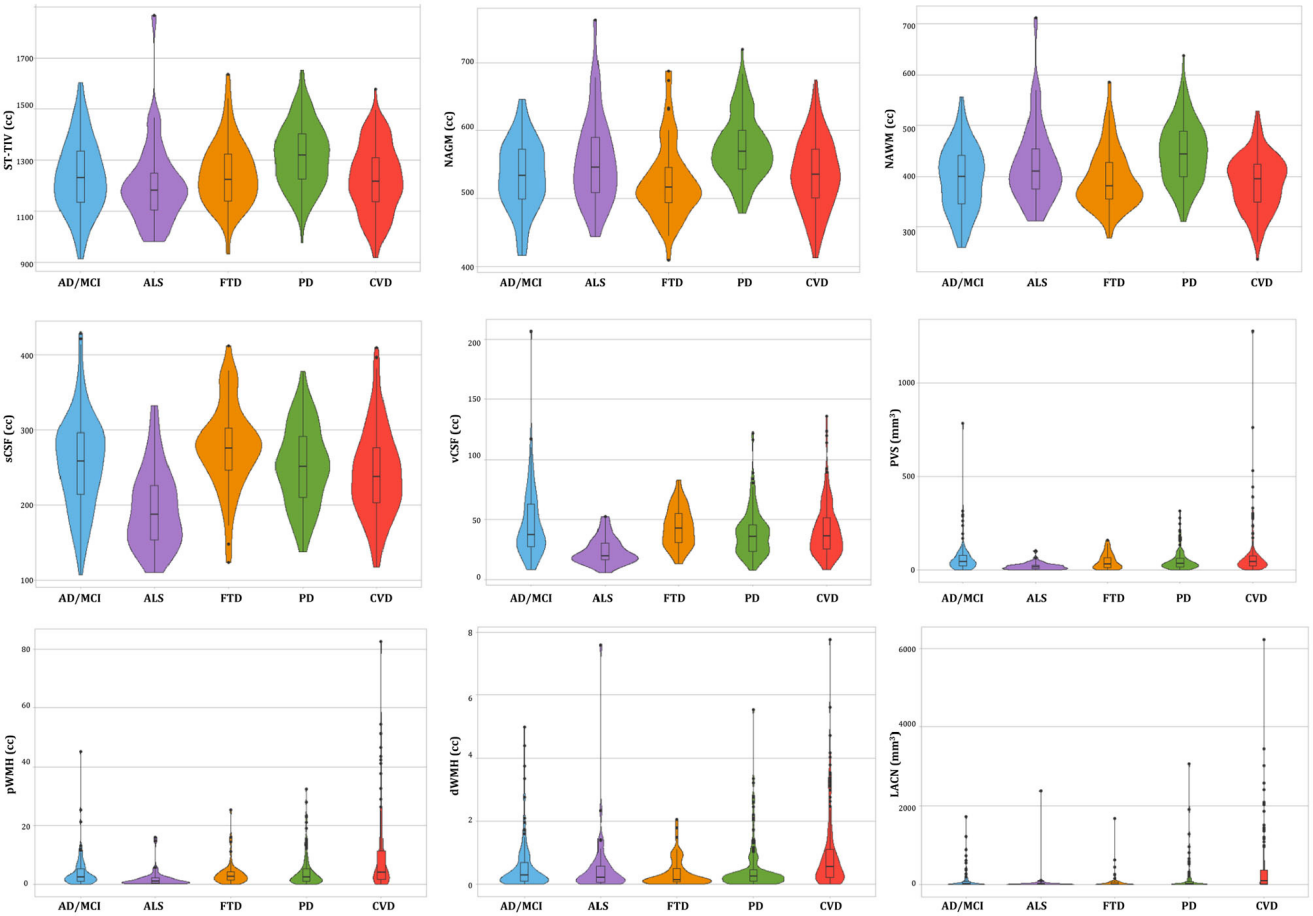
Descriptive violin plots of the ONDRI disease cohorts showing median and interquartile range volumetrics for whole brain supratentorial total intracranial volume (ST-TIV), normal appearing gray matter (NAGM), normal appearing white matter (NAWM), sulcal cerebrospinal fluid (sCSF), ventricular CSF (vCSF), MRI-visible enlarged perivascular space (PVS) volumes, periventricular white matter hyperintensities (pWMH), deep WMH (dWMH), and lacunes (LACN).

Of the *n* = 520 patients with MRI acquired, the ONDRI neuroimaging pipeline was unable to process *n* = 1 FTD patient due to extreme motion artifact (despite 2 baseline attempts on 2 separate occasions), and *n* = 6 CVD patients due to poor imaging quality (*n* = 3 FLAIR not usable, *n* = 2 T1 not usable, *n* = 1 PD/T2 not acquired). To illustrate whole brain data extraction volumetric results from this pipeline, neuroimaging summary statistics for each ONDRI disease cohort are summarized on Table 2, and descriptive violin plots showing median and interquartile ranges are provided for whole brain ST-TIV, NAGM, NAWM, sCSF, vCSF, pWMH, and dWMH PVS, and LACN are displayed on Figure 13. Stroke volumes were not graphed due to the limited number of ONDRI patients with cortico-subcortical stroke lesions.

It is important to note that the details in this manuscript focus on ONDRI's baseline data that will be released in October 2020, the longitudinal follow-up data will be forthcoming.

Additionally, a cohort of cognitively normal older adults recruited from the Brain-Eye Amyloid Memory (BEAM) study (clinicaltrials.gov—NCT02524405) with harmonized neuroimaging, neuropsychology, and data acquisition protocol, will be included in ONDRI for comparative analyses. Participants in BEAM were recruited from five sites (Sunnybrook, Baycrest, CAMH, SMH, and UHN) that also participated in ONDRI.

ONDRI is the first multi-site, multiple assessment platform study examining several neurodegenerative and neurovascular diseases using a harmonized protocol that includes standardized structural neuroimaging. The wide range of complex, and often overlapping, brain pathologies represented in this cohort of neurodegenerative patients included a number of comorbid cerebral small vessel disease markers, cortico-subcortical stroke lesions, combined with focal and global atrophy, posing significant challenges to common imaging analysis tools. In this paper, we presented the neuroimaging pipeline methods implemented in ONDRI that were used to overcome many of these challenges.

To further ensure a high level of data quality, the volumetric data generated by the ONDRI structural neuroimaging team were further subjected to comprehensive quality control analysis pipelines including a novel multivariate outlier detection algorithm developed by the ONDRI neuroinformatics group for identification of anomalous observations (114, 115). Future work will include generating longitudinal measures that will also be made publicly available. As the neuroimaging data are combined with releases from ONDRI's clinical, neuropsychology, genomics, eye tracking, gait and balance, ocular, and neuropathology platforms, it becomes evident that ONDRI is a gold mine of data opening the door to an unprecedented broad range of cross-platform analyses resulting in numerous opportunities for discovery and advances in diagnosis, prognosis, outcomes, and care of neurodegenerative diseases.

## Data Availability Statement

The datasets generated for this study are available on request to info@ondri.ca.

## Ethics Statement

The studies involving human participants were reviewed and approved by Ethics approval was obtained from all participating institutions. Participants were recruited at 14 health centers across six cities in Ontario, Canada: Hamilton General Hospital and McMaster Medical Centre in Hamilton; Hotel Dieu Hospital and Providence Care Hospital in Kingston; London Health Science Centre and Parkwood Institute in London; Elizabeth Bruyère Hospital and The Ottawa Hospital in Ottawa; Thunder Bay Regional Health Sciences Centre in Thunder Bay; and Baycrest Health Sciences, Centre for Addiction and Mental Health, St. Michael's Hospital, Sunnybrook Health Sciences Centre, and Toronto Western Hospital (University Health Network) in Toronto. The patients/participants provided their written informed consent to participate in this study.

## Author Contributions

JR: conceptualization, data curation, formal analysis, investigation, methodology, software, validation, visualization, writing (draft, review, and editing), and supervision. MH: data curation, formal analysis, investigation, methodology, software, validation, visualization, and writing (draft, review, and editing). CS: conceptualization, data curation, formal analysis, investigation, methodology, software, validation, visualization, writing (review and editing), and supervision. MO: data curation, methodology, software, visualization, and writing (review and editing). SA: data curation, software, validation, visualization, and writing (review and editing). GS: conceptualization, data curation, methodology, and software. MG: data curation, investigation, methodology, software, validation, and writing (draft, review, and editing). FG: conceptualization, data curation, investigation, validation, visualization, writing (review and editing), and supervision. SA and DB: data curation, formal analysis, investigation, validation, and writing (review and editing). JL-D: investigation, resources, validation, writing (draft, review, and editing), and funding acquisition. SCS: conceptualization, investigation, writing (draft, review, and editing), supervision, and funding acquisition. DM, MM, and RS: conceptualization, resources, investigation, writing (review and editing), supervision, and funding acquisition. RB: conceptualization, data curation, resources, investigation, writing (review and editing), and supervision. SS: data curation, resources, investigation, writing (review and editing), and supervision. SB: conceptualization, resources, investigation, methodology, visualization, writing (review and editing), supervision, and funding acquisition. All authors contributed to the article and approved the submitted version.

## Conflict of Interest

The authors declare that the research was conducted in the absence of any commercial or financial relationships that could be construed as a potential conflict of interest.

## ONDRI Investigators

Michael Strong, Peter Kleinstiver, Natalie Rashkovan, Susan Bronskill, Sandra E. Black, Michael Borrie, Elizabeth Finger, Corinne Fischer, Andrew Frank, Morris Freedman, Sanjeev Kumar, Stephen Pasternak, Bruce Pollock, Tarek Rajji, Dallas Seitz, David Tang-Wai, Carmela Tartaglia, Brenda Varriano, Agessandro Abrahao, Marvin Chum, Christen Shoesmith, John Turnbull, Lorne Zinman, Jane Lawrence-Dewar, Donna Kwan, Brian Tan, Julia Fraser, Bill McIlroy, Ben Cornish, Karen Van Ooteghem, Frederico Faria, Manuel Montero-Odasso, Yanina Sarquis-Adamson, Alanna Black, Barry Greenberg, Wendy Hatch, Chris Hudson, Elena Leontieva, Ed Margolin, Efrem Mandelcorn, Faryan Tayyari, Sherif Defrawy, Don Brien, Ying Chen, Brian Coe, Doug Munoz, Alisia Bonnick, Leanne Casaubon, Dar Dowlatshahi, Ayman Hassan, Jennifer Mandzia, Demetrios Sahlas, Gustavo Saposnik, Richard H. Swartz, David Breen, David Grimes, Mandar Jog, Anthony Lang, Connie Marras, Mario Masellis, Tom Steeves, Dennis Bulman, Allison Ann Dilliott, Mahdi Ghani, Rob Hegele, John Robinson, Ekaterina Rogaeva, Sali Farhan, Rob Bartha, Hassan Haddad, Nuwan Nanayakkara, Joel Ramirez, Christopher Scott, Sean Symons, Courtney Berezuk, Melissa Holmes, Sabrina Adamo, Miracle Ozzoude, Mojdeh Zamyadi, Stephen Arnott, Derek Beaton, Malcolm Binns, Wendy Lou, Pradeep Raamana, Stephen Strother, Kelly Sunderland, Athena Theyers, Abiramy Uthirakumaran, Guangyong (GY) Zou, Sujeevini Sujanthan, Mojdeh Zamyadi, David Munoz, Roger A. Dixon, John Woulfe, Brian Levine, Paula McLaughlin, JB Orange, Alicia Peltsch, Angela Roberts, Angela Troyer.
