# Supplementary material for: Ontario Neurodegenerative Disease Research Initiative (ONDRI): Structural MRI Methods and Outcome Measures
Source: Front Neurol. 2020 Aug 11;11:847. doi: 10.3389/fneur.2020.00847 (PMC7431907; doi:10.3389/fneur.2020.00847)
Supplement: Supplementary file 1 [file Data_Sheet_1.pdf]

Supplementary Table 1 - MRI Acquisition Protocols

STUDY	OBI - ONDRI		
SEQUENCE	3DT1		
**Protocol**			
Vendor	GE	Philips	Siemens
Field Strength	3T	3T	3T
Model	Discovery	Achieva	Skyra/Trio/Prisma
Version	22	3.2.3	
Sequence Name	3D FAST SPGR	3D TFE	3D MP-RAGE
Imaging Options	IrP- Asset	Fast (Sense)	iPat
**Pulse Timing**			
TE (ms)	Min full	Min (3.3)	2.98
TR (ms)	Min	Min (7.3)	2300
Flip Angle (°)	11	9	9
TI (ms)	400	945	900
**Scan Range**			
FOV (in-plane) (mm)	256 x 256	256 x 248	256 x 256
Slice Thickness (mm)	1	1	1
Gap Between Slices (mm)	0	0	0
No. Slices	176	176	176
**Acquisition**			
Orientation	Sagittal	Sagittal	Sagittal
Matrix Size	256 x 256	256 x 248	256 x 256
Voxel Size [L/R x A/P x I/S]	1 x 1 x 1	1 x 1 x 1	1 x 1 x 1
NEX	1	1	1
Acceleration Factor (Parallel factor*)	2	2	2
**Other**			
Fat Suppression	None	None	None
Bandwidth	31.25 (kHz)	228 (Hz/px)	240 (Hz/px)
Echo Train Length	-	-	-
**Coil Type**			
Head	X	X	X
Channel	8-12 (HNS)	8	12(Trio) 20 (Prisma)
**Time**			
PRESCAN TIME+	00:30	00:30	00:30
SCAN TIME	04:52	06:17	05:21
TOTAL TIME (MIN)	05:22	06:47	05:51

Study	***OBI - ONDRI***		
Sequence	***PD/T2***		
Protocol			
Vendor	GE	Philips	Siemens
Field Strength	3T	3T	3T
Model	Discovery	Achieva	Skyra/Trio/Prisma
Version	22	3.2.3	
Sequence Name	FSE-XL	TSE	TSE
Imaging Options	EDR, Asset	Fast (Sense)	iPat
Pulse Timing			
TE (ms) (2 echo scan)	Min full/86	13/100	10/93
TR (ms)	3000	3000	3000
Flip Angle (°)	125	90	165
TI (ms)	-	-	-
Scan Range			
FOV (in-plane) (mm)	240 x 240	240 x 240	240 x 240
Phase FOV	75%	75%	81%
Slice Thickness (mm)	3	3	3
Gap Between Slices (mm)	0	0	0
No. Slices	48	48	48
Acquisition			
Orientation	Oblique Axial	Oblique Axial	Oblique Axial
Matrix Size	256 x 256	256 x 254	256 x 256
Voxel Size [L/R x A/P x I/S]	0.94 x 0.94 x 3	0.94x 0.94 x 3	0.94 x 0.94 x 3
NEX	1	1	1
Acceleration Factor (Parallel factor*)	2	2	2
Other			
Fat Suppression	Yes (FAT-SAT)	Yes	Yes
Bandwidth	20 (kHz)	222 (Hz/px)	181 (Hz/px)
Echo Train Length	12	12	14
Coil Type			
Head	X	X	X
Channel	8-12 (HNS)	8	12(Trio) 20 (Prisma)
Time			
Prescan Time+	00:30	00:30	00:30
Scan Time	02:43	04:12	03:11
Total Time (min)	03:13	04:42	03:41

Study	***OBI - ONDRI***		
Sequence	***2D FLAIR***		
Protocol			
Vendor	GE	Philips	Siemens
Field Strength	3T	3T	3T
Model	Discovery	Achieva	Skyra/Trio/Prisma
Version	22	3.2.3	
Sequence Name	2D T2FLAIR	2D IR TSE	2D IR TDF
Imaging Options	EDR, IR	Fast (Sense)	iPat
Pulse Timing			
TE (ms)	140	125	120
TR (ms)	9000	9000	9000
Flip Angle (°)	125	90 (150 refocus)	165
TI (ms)	2250	2500	2500
Scan Range			
FOV (in-plane) (mm)	240 x 240	240 x 240	240 x 240
Slice Thickness (mm)	3	3	3
Gap Between Slices (mm)	0	0	0
No. Slices	48	48	48
Acquisition			
Orientation	Oblique Axial	Oblique Axial	Oblique Axial
Matrix Size	256 x 256	256 x 242	256 x 256
Voxel Size [L/R x A/P x I/S]	0.94 x 0.94 x 3	0.94 x 0.99 x 3	0.94 x 0.94 x 3
NEX	1	1	1
Acceleration Factor (Parallel factor*)	No Asset	2 (SENSE)	2
Other			
Fat Suppression	None	None	None
Bandwidth	25 (kHz)	242 (Hz/px)	220 (Hz/px)
Echo Train Length		19	19
Coil Type			
Head	X	X	X
Channel	8-12 (HNS)	8	12(Trio) 20 (Prisma)
Time			
Prescan Time+	00:30	00:30	00:30
Scan Time	04:32	03:45	02:44
Total Time (min)	05:02	04:15	03:14

STUDY	OBI - ONDRI		
SEQUENCE	T2-star		
**Protocol**			
Vendor	GE	Philips	Siemens
Field Strength	3T	3T	3T
Model	Discovery	Achieva	Skyra/Trio/Prisma
Version	22	3.2.3	
Sequence Name	GRE	FFE	GRE
Imaging Options	-	Sense	iPat
**Pulse Timing**			
TE (ms)	20	21	20
TR (ms)	650	650	650
Flip Angle (°)	20	20	20
TI (ms)	-	-	-
**Scan Range**			
FOV (in-plane) (mm)	240 x 240	240 x 240	240 x 240
Phase FOV	75%	75%	75%
Slice Thickness (mm)	3	3	3
Gap Between Slices (mm)	0	0	0
No. Slices	48	48	48
**Acquisition**			
Orientation	Oblique Axial	Oblique Axial	Oblique Axial
Matrix Size	256 x 256	256 x 256	256 x 256
Voxel Size [L/R x A/P x I/S]	0.94 x 0.94 x 3	0.94 x 0.94 x 3	0.94 x 0.94 x 3
NEX	1	1	1
Acceleration Factor (Parallel factor*)	No Asset	2 (SENSE)	2
**Other**			
Fat Suppression	None	None	None
Bandwidth	19.23 (kHz)	217 (Hz/px)	200 (Hz/px)
Echo Train Length	1	1	1
CV act_te (GE only)	20000		
**Coil Type**			
Head	X	X	X
Channel	8-12 (HNS)	8	12(Trio) 20 (Prisma)
**Time**			
PRESCAN TIME+	00:30	00:30	00:30
SCAN TIME	02:15	02:52	03:04
TOTAL TIME (MIN)			

STUDY	OBI - ONDRI		
SEQUENCE	fMRI-RS		
**Protocol**			
Vendor	GE	Philips	Siemens
Field Strength	3T	3T	3T
Model	Discovery	Achieva	Skyra/Trio/Prisma
Version	22	3.2.3	
Sequence Name	fMRI EPI	fMRI EPI	fMRI EPI
Imaging Options	EDR, "eyes open"	GRE EPI, CLEAR SENSE, "eyes open"	"eyes open"
**Pulse Timing**			
TE (ms)	30	30	30
TR (ms)	2400	2400	2400
Flip Angle (°)	70	70	70
TI (ms)	-	-	-
**Scan Range**			
FOV (in-plane) (mm)	224 x224	224 x 224	224 x 224
Slice Thickness (mm)	3.5	3.5	3.5
Gap Between Slices (mm)	0	0	0
No. Slices	41	41	41
**Acquisition**			
Orientation	Oblique Axial	Oblique Axial	Oblique Axial
Matrix Size	64 x 64	64 x 64	64 x 64
Voxel Size [L/R x A/P x I/S]	3.5 x 3.5 x 3.5	3.5 x 3.5 x 3.5	3.5 x 3.5 x 3.5
NEX	1	1	1
Acceleration Factor (Parallel factor*)	2	2	2
Slice Order		Ascending	
**Other**			
Fat Suppression	Fat Sat.	Fat Sat. SPIR	Fat Sat.
Bandwidth (Hz/Px)	7812	2441	2440
Number of acquisitions	250	250	250
EPI Factor		35	31
**Coil Type**			
Head	X	X	X
Channel	8-12 (HNS)	8	12(Trio) 20 (Prisma)
**Time**			
PRESCAN TIME+	00:30	00:30	00:30
SCAN TIME	10:00	10:00	10:00
TOTAL TIME (MIN)	10:30	10:30	10:30

Study	***OBI - ONDRI***			
Sequence	***DTI***			
Protocol				
Vendor	GE	Philips	Siemens	Siemens
Field Strength	3T	3T	3T	3T
Model	Discovery	Achieva	Skyra/Trio/Prisma	Skyra/Trio/Prisma
Version	22	3.2.3		17
Sequence Name	DWI	DWI	DWI	DWI
Imaging Options	ASSET	Sense	iPat	iPat
Pulse Timing				
TE (ms)	Min	100	96/ 63(Prisma)	96/ 64 (Prisma)
TR (ms)	9000	9931	9400	9400
Flip Angle (°)	90	90	90	90
TI (ms)	-	-	-	-
Scan Range				
FOV (in-plane) (mm)	256 x 256	256x 256	256 x 256	256 x 256
Slice Thickness (mm)	2	2	2	2
Gap Between slices (mm)	0	0	0	0
No. Slices	70	70	70	70
Acquisition				
Orientation	Oblique Axial	Oblique Axial	Oblique Axial	Oblique Axial
Matrix Size	128 x 128	128 x 128	128 x 128	128 x 128
Voxel Size (L/R x A/P x I/S)	2 x 2 x 2	2 x 2 x 2	2 x 2 x 2	2 x 2 x 2
NEX	1	1	1	1
Acceleration Factor (Parallel factor*)	2	2	2	2
Diffusion				
b-value 1	0	0	0	0
b-value 2	1000	1000	1000	
Number of Directions	30	32	30	1
Other				
Fat Suppression	FatSat	FatSat	FatSat	FatSat
Bandwidth (Hz/Px)		2045	2056	2056
T2 Images	3	3	3	
EPI Factor		67	128	128
Gradients			Monopolar (Prisma)	Monopolar (Prisma)
CV rhimsize (GE only)	128			
Coil Type				

Head	x	x	x	x
Channel	8-12 (HNS)	8	12(Trio) 20 (Prisma)	12(Trio) 20 (Prisma)
Time				
**Prescan Time+**	00:30	00:30	00:30	00:30
**Scan Time**	05:06	0:6:58	05:21	00:39
**Total Time (min)**	05:36	07:28	05:33	01:08

Core Protocol Scan Time Estimates

	GE	*Philips*	*Siemens*
Set up	*05:00*	*05:00*	*05:00*
3DT1	05:22	06:47	05:51
PD/T2	02:49	04:42	03:41
2D FLAIR	05:02	04:15	03:14
T2-star *or* T2-star_PM *or* T2-star_PMRI	03:45	03:22	03:34
RS-fMRI	10:30	10:30	10:30
DTI	06:30	07:28	06:41
Set down	*02:30*	*02:30*	*02:30*
**Total scan time**	33:58	37:04	33:31
**Total session time**	**41:28**	**44:34**	**41:01**
